# Bi_5_O_7_I/g-C_3_N_4_ Heterostructures With Enhanced Visible-Light Photocatalytic Performance for Degradation of Tetracycline Hydrochloride

**DOI:** 10.3389/fchem.2021.781991

**Published:** 2021-12-14

**Authors:** Yang Yang, Min Lai, Jialei Huang, Jinze Li, Ruijie Gao, Ziming Zhao, Huatang Song, Jixiang He, Yan Ma

**Affiliations:** ^1^ Jiangsu Key Laboratory for Optoelectronic Detection of Atmosphere and Ocean, Nanjing University of Information Science and Technology, Nanjing, China; ^2^ School of Physics and Optoelectronic Engineering, Nanjing University of Information Science and Technology, Nanjing, China; ^3^ NUIST Reading Academy, Nanjing University of Information Science and Technology, Nanjing, China

**Keywords:** photocatalysis, heterostructures, visible light adsorption, charge carrier separation, tetracycline hydrochloride

## Abstract

Bi_5_O_7_I/g-C_3_N_4_ p-n junctioned photocatalysts were synthesized by alcohol-heating and calcination in air. The structures, morphologies and optical properties of as-prepared samples were characterized by X-ray diffraction (XRD), scanning electron microscopy (SEM), transmission electron microscopy (TEM), UV–Vis diffuse reflectance spectroscopy (DRS). Photocatalytic activity of the heterojunctioned composites were evaluated by degradation of Rhodamine B (RhB) and tetracycline hydrochloride (TCH) under visible light illumination. The results indicated that the composites exhibited superior efficiencies for photodegradation of RhB and TCH in comparison with pure BiOI, Bi_5_O_7_I and g-C_3_N_4_. An effective built-in electric field was formed by the interface between p-type Bi_5_O_7_I and n-type g-C_3_N_4_, which promoted the efficient separation of photoinduced electron-hole pairs. In addition, 8% Bi_5_O_7_I/g-C_3_N_4_ composite showed excellent photostability in a five-cycle photocatalytic experiment. Experiments on scavenging active intermediates revealed the roles of active species.

## Introduction

Tetracycline antibiotics have a wide range of applications as broad-spectrum antibiotics. TCH with a high water solubility is formed by combining tetracycline with hydrochloric acid, which is widely used for treatment of human and animal diseases owing to its low cost, broad antibacterial spectrum, high chemical stability and low side effects (Wang and Wang, 2015). However, TCH has been overused worldwide in animal husbandry and aquaculture production. It is difficult to achieve total removal of TCH in natural environment due to its benzene-containing skeleton, which results in its long-term existence in water environment and harm to ecological environment and human health. Among various treatment methods of TCH, photocatalytic oxidation technology exhibits advantage of environmental-friendly process, low-energy cost and easy operation (Chen and Liu, 2016).

In recent years, semiconductor-based photocatalysts have been developed intensively due to their potential applications in water splitting (Kudo and Miseki, 2003), degradation of organic pollutants (Carey et al., 1976), photocatalytic reduction of carbon dioxide (Roy et al., 2010), photocatalytic organic synthesis (Xiao et al., 2015), etc. Titanium dioxide as a traditional semiconducting photocatalyst, possesses a band gap energy of about 3.2 eV (Schneider et al., 2014). The large band gap results in limited response in ultraviolet light range which accounts for only about 4% in the whole energy of sunlight. Furthermore, in these photocatalysts, photogenerated electron-hole pairs are easy to recombine, which reduces the efficiency of photocatalysis. Various photocatalytic systems have been developed to enhance catalytic performance in degradation of organic pollutants in the past decades. Among these methods, formation of heterostructures has shown a promising improvement in the efficiency of charge separation and transfer.

BiOI is a p-type of photocatalytic material with a layered structure. It has attracted intense scientific interest due to its unique properties based on narrow band gap and excellent photocatalytic performance (Lei et al., 2010; Rong et al., 2012; Chang et al., 2019). Bi_5_O_7_I is an n-type semiconductor with a high thermodynamic stability. Due to the decrease in proportion of iodine atoms, the positions of conduction and valence bands of Bi_5_O_7_I vary from BiOI, which has been extensively studied as a photocatalyst in degradation of organic pollutants (Geng et al., 2018; Wang et al., 2018; Xia et al., 2018). Bi_5_O_7_I possesses a more negative valence potential, which enables it to generate more holes to degrade pollutants after photoactivation (Liang et al., 2019). However, pure Bi_5_O_7_I is limited for application in photocatalysis because of weak visible-light absorption and easy recombination of photo-generated charge carriers. Formation of a heterojunctioned structure by combining Bi_5_O_7_I with other suitable semiconductors is possibly a promising method to improve the photocatalytic activity. g-C_3_N_4_ is an n-type of non-metallic photocatalyst with excellent optical absorption in the visible light range, which also shows good thermal stability, chemical stability and optical stability. Its band gap of about 2.7 eV has led to a good candidate to form heterojunctioned photocatalysts including TiO_2_/C_3_N_4_ (Yan et al., 2016), BiOBr/g-C_3_N_4_ (Ye et al., 2013), g-C_3_N_4_/Bi_2_WO_6_ (Li et al., 2015a) and graphene/C_3_N_4_ (Xiang et al., 2011). Liu et al. (2015) prepared a Bi_5_O_7_I based g-C_3_N_4_/Bi_5_O_7_I composite photocatalyst *via* an *in situ* co-crystallization route, which exhibited enhanced photocatalytic activity for degradation of RhB and phenol under visible light.

Efficient degradation of TCH remains a challenge for semiconductor-based photocatalysis (Chen and Liu, 2016). In this work, a facile synthesis of Bi_5_O_7_I/g-C_3_N_4_ heterojunctioned structures were developed to enhance absorption of visible light and separation of photoinduced electron-hole pairs. Bi_5_O_7_I/g-C_3_N_4_ heterostructures were formed by synthesis of BiOI via an alcohol-heating route followed by calcination after mixing with urea powder. Photocatalytic performance of the heterojunctioned composites were evaluated by degradation of RhB and TCH under visible light illumination.

## Materials and Methods

### Synthesis of Bi_5_O_7_I/g-C_3_N_4_ Composites

All the chemicals were analytical grade and used as received without further purification. BiOI powders were synthesized according to the previous report (Xiang et al., 2016). Briefly, Bi (NO_3_)_3_·5H_2_O (AR, 99.0%) was added slowly into an ethylene glycol (EG) solution containing KI (AR, 99.0%) with the Bi/I molar ratio of 1. The mixture was stirred for 0.5 h at room temperature in air, and then poured into a 50 ml Teflon-lined stainless autoclave until 80% of the autoclave volume was filled. The autoclave was allowed to be heated at 160°C for 12 h under autogenous pressure, and was then cooled to room temperature in air. The resulting precipitates were collected and washed with ethanol and deionized water and dried at 60°C in air for 12 h.

Bi_5_O_7_I/g-C_3_N_4_ heterojunctioned composites were synthesized by a calcination method. BiOI and urea powers were mixed and ground for 0.5 h with agate grinding bowl. The resulting solid was placed in a crucible covered with an alumina lid and was then heated to 500°C in a muffle furnace for 2 h with a heating rate of 15°C/min. After cooling naturally to room temperature, the pale yellow resultant was washed for three times with deionized water and ethyl alcohol, respectively, and dried at 60°C in air for 12 h. The product was labeled as x% BiOI/g-C_3_N_4_ where x% is the mass percentage of BiOI to urea. Sample 8% BiOI/g-C_3_N_4_ was calcined and washed again by the same process. The final product was labeled as 8% Bi_5_O_7_I/g-C_3_N_4_. For comparison on photocatalytic performance, pure Bi_5_O_7_I or g-C_3_N_4_ samples were synthesized under the same condition without addition of urea or BiOI.

### Characterization

X-ray diffraction (XRD) patterns of the samples were carried out on a diffractometer XRD-6100 (Shimadzu, Japan) operating at 40 KV and 30 mA with Cu *K*_α_ radiation (λ = 0.154,056 nm). Morphology of the samples was observed using a field-emission scanning electron microscope (FE-SEM) SU-1510 (Hitachi, Japan) and a high-resolution transmission electron microscope (HRTEM) Tecnai G2 F20 (FEI Company, United States). Reflection spectra of the samples were measured on a UV-Vis-NIR spectrophotometer UV-3600 (Shimadzu, Japan) in the range of 260–750 nm and BaSO_4_ was employed as a reflectance. Brunauer–Emmett–Teller (BET) specific surface areas of the samples were analyzed by Autosorb-IQ-C specific surface analyzer (Kanta Instruments, United States). Chemical oxygen demand (COD) was evaluated by COD Max II Chemical Oxygen Demand Velocity Tester (Hach Company, United States). A CHI 660E electrochemical workstation and a three-electrode system including a reference electrode (RE), a counter electrode (CE) and a working electrode (WE) were used in photocurrent tests. The counter and reference electrodes were a piece of Pt mesh and a saturated calomel electrode, respectively. The working voltage was a positive bias of 1 V, the light source was a 20 W LED lamp, and the illumination interval was 20 s.

### Photocatalytic Activity Tests

Degradation of RhB and TCH under visible light was used to evaluate the photocatalytic activities of all as-prepared samples. A 300 W xenon lamp equipped with a 420 nm cutoff filter severed as a visible light source was placed 15 cm above the water bath. Water circulation system was used to keep reactant suspension at 20°C avoiding the influence of photo-thermal effect. In a typical experiment, a suspension was prepared by mixing 0.1 g photocatalyst into 100 ml of 8 mgL^−1^ RhB aqueous solution and 100 ml of 20 mgL^−1^ TCH aqueous solution, respectively. Before illumination, the suspension was vigorously stirred in the dark for 1 h to reach adsorption-desorption equilibrium. At given irradiation time intervals of 15 min, 3 ml of suspensions were collected and centrifuged at 5,000 rpm for 3 min to remove the catalysts. The absorbance of the solution was analyzed at 554 nm for RhB and 357 nm for TCH by UV-Vis spectrophotometer to monitor photocatalytic degradation of RhB and TCH, respectively.

## Results and Discussion

### XRD Analysis

**FIGURE 1:**
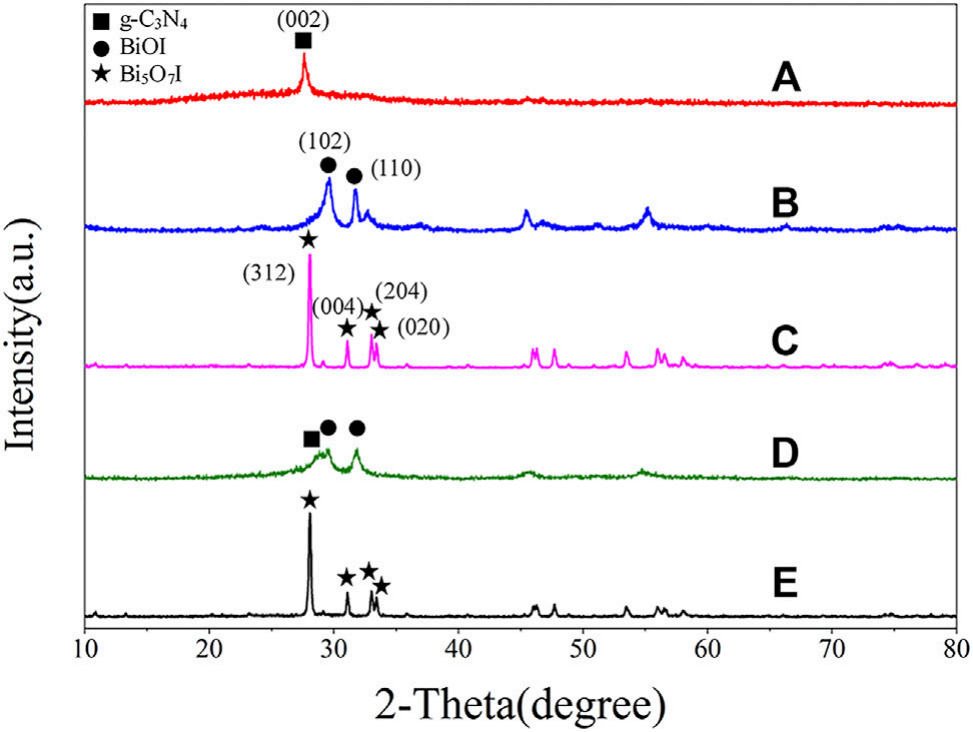
XRD patterns of **(A)** g-C3N4, **(B)** BiOI, **(C)** Bi5O7I, **(D)** 8% BiOI/g-C3N4, **(E)** 8% Bi5O7I/g-C3N4.

XRD was performed to investigate the phase structures of as-prepared samples and the results of g-C_3_N_4_, BiOI, Bi_5_O_7_I, BiOI/g-C_3_N_4_ and Bi_5_O_7_I/g-C_3_N_4_ are shown in Figure 1. The strongest peak of pure g-C_3_N_4_ (JCPDS No.87-1,526) at 27.75° is assigned to its (002) crystal plane (Yang et al., 2013; Dong et al., 2015; Ho et al., 2015). The characteristic diffraction peaks at 2θ = 29.64° and 31.65° for pure BiOI, correspond to the (102) and (110) crystal planes of tetragonal BiOI, respectively (JCPDS, No.10-0445). The diffraction peaks at 28.06°, 31.08°, 33.02°, and 33.40° observed on pure Bi_5_O_7_I are indexed to crystal planes (312), (004), (204) and (020) of orthorhombic Bi_5_O_7_I, respectively, according to the XRD standard card (JCPDS No.40-0548), which suggested the formation of Bi_5_O_7_I after calcination for twice at 500°C. The XRD pattern of 8% BiOI/g-C_3_N_4_ presents characteristic diffraction peaks corresponding to BiOI and g-C_3_N_4_, indicating formation of composite including BiOI and g-C_3_N_4_ after the first calcination at 500°C. All the peaks in the XRD pattern of 8% Bi_5_O_7_I/g-C_3_N_4_ were indexed to the characteristic diffraction of crystal planes of orthorhombic Bi_5_O_7_I, suggesting that BiOI has been transformed into Bi_5_O_7_I after the second calcination at 500°C. No obvious characteristic diffraction peaks were identified for g-C_3_N_4_ in the sample, which can be ascribed to low crystallinity of g-C_3_N_4_ and overlap of the main peaks with Bi_5_O_7_I at 28.06° and g-C_3_N_4_ at 27.75°. In addition, no impurity peaks are detected in these patterns, revealing that the composites are composed of g-C_3_N_4_ and Bi_5_O_7_I with a high purity.

### SEM and BET Analysis

**FIGURE 2:**
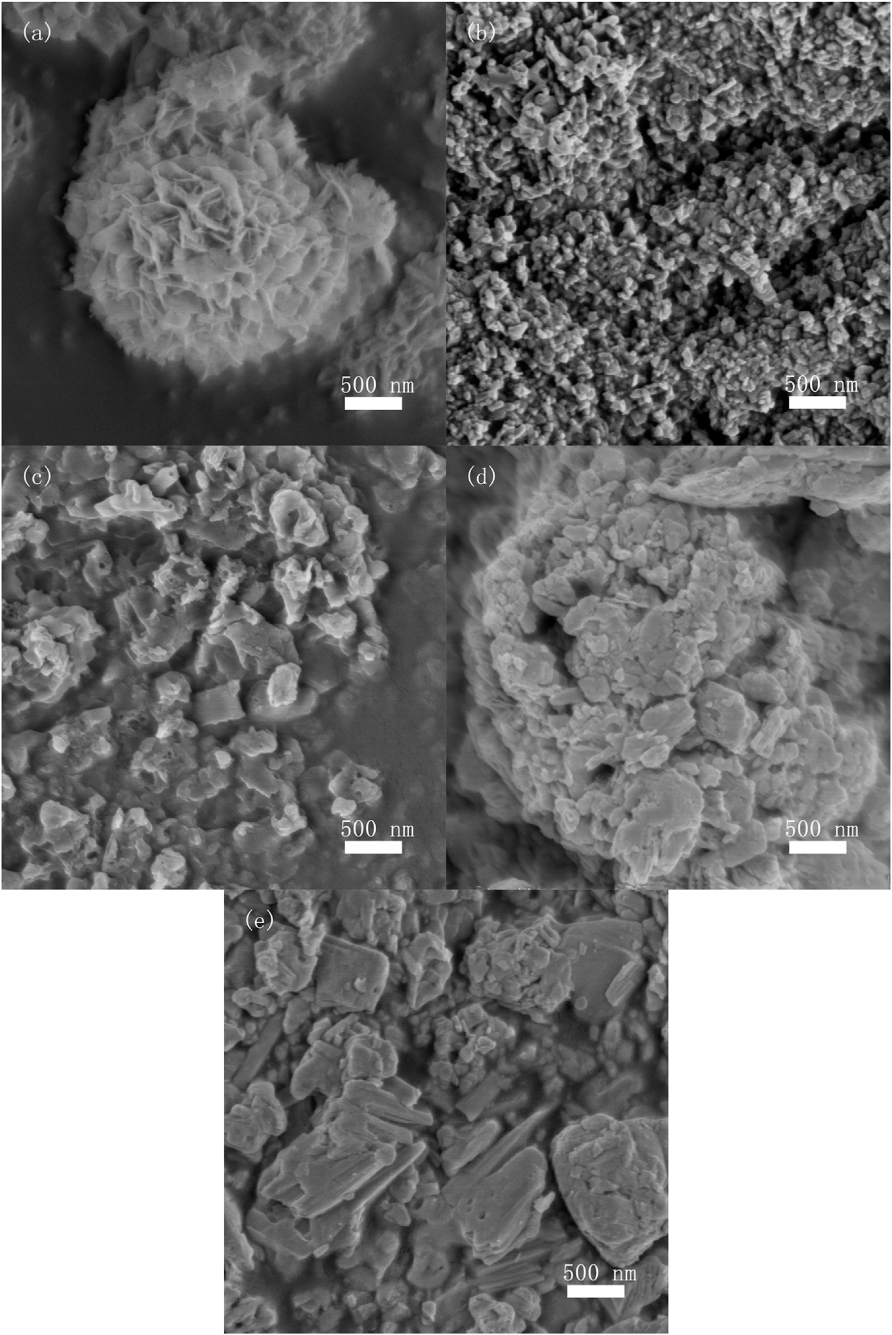
FE-SEM images of **(A)** BiOI, **(B)** Bi5O7I, **(C)** bulk g-C3N4, **(D)** 8% BiOI/g-C3N4, **(E)** 8%Bi5O7I/g-C3N4.

Figure 2 shows typical FE-SEM images of as-prepared photocatalysts. Microspherical structures with diameter of about 2.5 μm, composed of nanosheets, were observed in BiOI sample, as shown in Figure 2A. Figure 2B shows the aggregated particles of Bi_5_O_7_I photocatalyst which was formed by calcination of BiOI in air. Figures 2D,E show the aggregation of micrometer sized irregular particles of BiOI/g-C_3_N_4_ and Bi_5_O_7_I/g-C_3_N_4_ composites, respectively. In the specific surface analysis testing, the specific surface areas of Bi_5_O_7_I, g-C_3_N_4_ and 8% Bi_5_O_7_I/g-C_3_N_4_ were determined to be 17.6, 14.2 and 16.5 m^2^g^−1^, respectively which indicated that specific surface area had a trivial effect on photocatalytic performance.

### TEM Analysis

**FIGURE 3:**
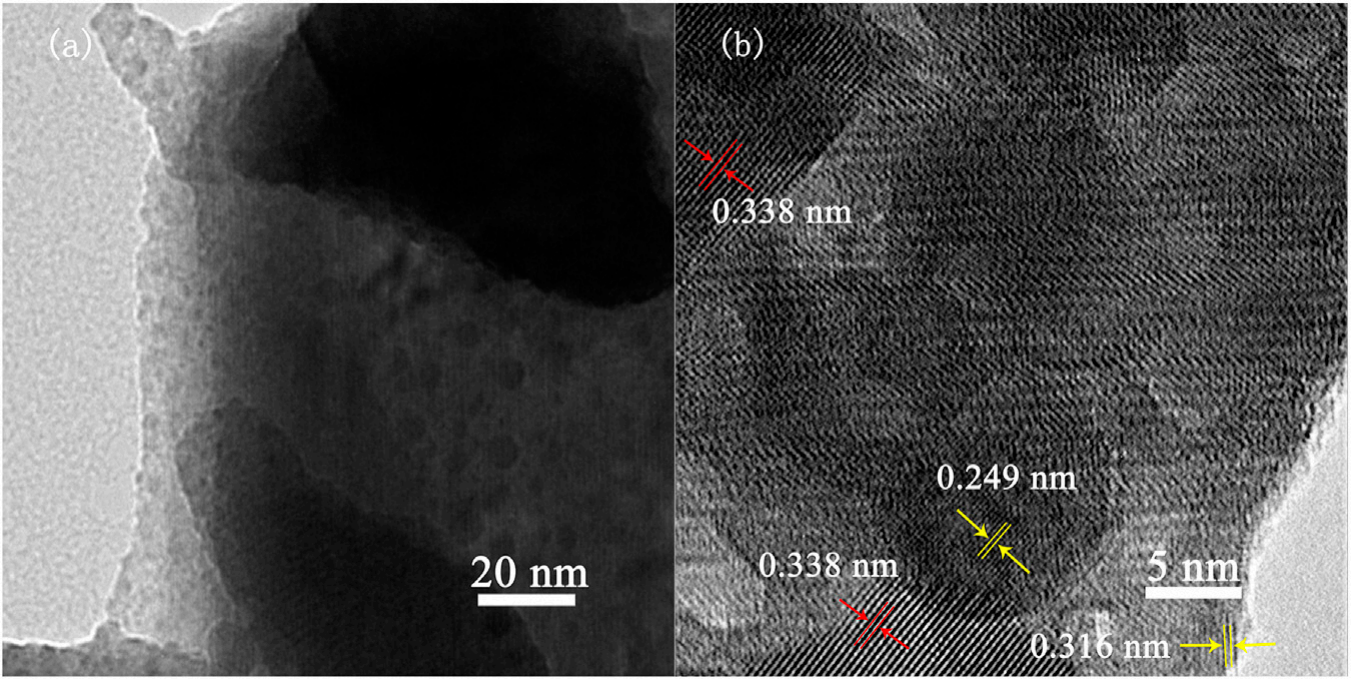
**(A)** TEM and **(B)** HRTEM images of 8% Bi5O7I/g-C3N4.

Sample 8% Bi_5_O_7_I/g-C_3_N_4_ was further characterized by TEM, as shown in Figure 3. Nanoparticles with diameter of several nanometers were distributed in the composite, suggesting that the as-synthesized Bi_5_O_7_I/g-C_3_N_4_ composite was possibly mixtures of two different components. In the HRTEM image (Figure 3B), the lattice fringes of 0.338, 0.249 and 0.316 nm coincide with the interplanar spacing of plane (002) of g-C_3_N_4_ and planes (114) and (312) of Bi_5_O_7_I, respectively, which is consistent with the results of XRD analysis. Furthermore, Bi_5_O_7_I nanospheres were distributed on the surface of g-C_3_N_4_, which implied a superior absorption of visible light. Based on the results of XRD, SEM, and TEM, the composites were successfully synthesized and formation of the interfaces between Bi_5_O_7_I and g-C_3_N_4_ confirmed a p-n junction structure in the composite.

### UV-Vis DRS Analysis

**FIGURE 4:**
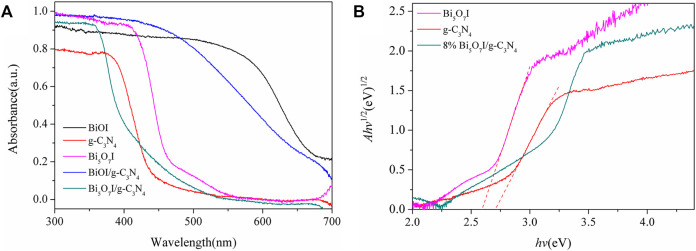
**(A)** UV–vis diffuses reflectance spectra and **(B)** (*Ahv*)^1/2^versus *hv*plots ofg-C3N4, Bi5O7Iand 8% Bi5O7I/g-C3N4.

Figure 4 exhibits the UV-Vis diffuse absorbance spectra of as-prepared photocatalysts. Apparently, BiOI absorbs light most effectively with an absorption edge at about 700 nm. Comparatively, the pure g-C_3_N_4_ sample exhibits a shorter absorption edge, which is located at about 450 nm. The light absorption threshold of samples extended to longer wavelength with the introduction of BiOI and Bi_5_O_7_I, which indicated that the visible light adsorption of as-prepared composites was contributed by the formation of BiOI and Bi_5_O_7_I. The band gap energies of as-prepared composites can be calculated using classical Tauc’s approach

$$ahv=A{\left(\mathrm{hv}-E_{g}\right)}^{n/2}$$ (1)

where *α*, *h*, *υ*, A and *E*_g_, are absorption coefficient, Planck’s constant, light frequency, proportionality constant and band gap energy, respectively (Huang et al., 2014). Among them, values of *n* are 4 for indirect transition and 1 for direct transition, respectively (Xu et al., 2015). Since both Bi_5_O_7_I and g-C_3_N_4_ are indirect transition semiconductors (Yang et al., 2014; Liu et al., 2015), values of *n* are 4. Hence, values of *E*_g_ for Bi_5_O_7_I and g-C_3_N_4_ can be estimated to be 2.59 and 2.71 eV respectively. These *E*_g_ values are in accordance with those in previous reports (Chen et al., 2015; Bai et al., 2016).

### Photocatalytic Properties

**FIGURE 5:**
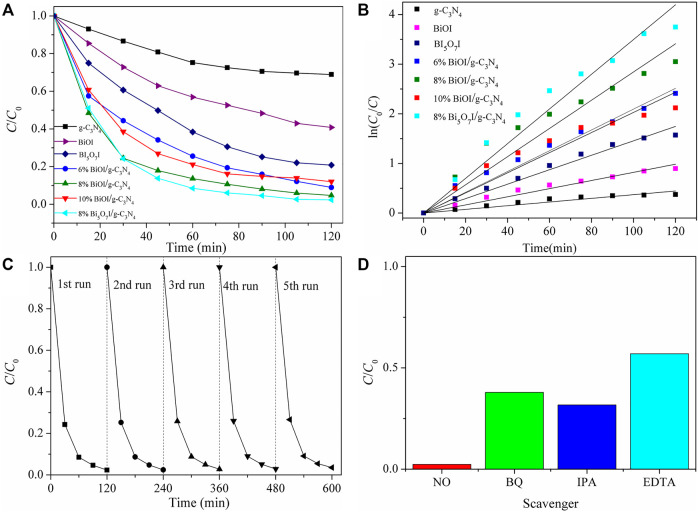
**(A)** Photocatalytic degradation of RhB byas-prepared samples under visiblelight irradiation, **(B)** corresponding ln (*C*0*/C*) versus time of the degradation reaction, **(C)** cycling runs in the photocatalytic degradation of RhB with 8% Bi5O7I/g-C3N4, **(D)** influence of different scavengers on degradation of RhB with 8% Bi5O7I/g-C3N4under visible light illumination after 120 min.

The photocatalytic activities of as-synthesized samples were first characterized by degradation of RhB under visible light illumination (≥420 nm), as shown in Figure 5A. *C* was the concentration of RhB during the reaction and *C*_0_ was the initial concentration of RhB. As for pure g-C_3_N_4_ and BiOI, only 21 and 39% of RhB were photodegraded after visible-light irradiation for 120 min, respectively. Meanwhile, Bi_5_O_7_I achieved superior photocatalytic activity over BiOI. After formation of BiOI/g-C_3_N_4_ and Bi_5_O_7_I/g-C_3_N_4_, the composites show a significant increase in RhB degradation performance compared with the pure BiOI, Bi_5_O_7_I and g-C_3_N_4_. The degradation of RhB was fitted for first-order kinetics with a Langmuir-Hinshelwood model (Sun et al., 2014): ln (*C*_0_ */C*) *= kt-*ln (*C*_0_ */C*_1_), where *k* is the reaction rate constant, which were presented in Figure 5B. The reaction rate constants of pure g-C_3_N_4_, pure BiOI and pure Bi_5_O_7_I were estimated to be 0.00370 min^−1^, 0.00820 min^−1^, 0.01456 min^−1^, respectively. With formation of composites, the *k* values increased to 0.02095 min^−1^, 0.02843 min^−1^ 0.02028 min^−1^ and 0.03497 min^−1^ for 6% BiOI/g-C_3_N_4_, 8% BiOI/g-C_3_N_4_, 10% BiOI/g-C_3_N_4_ and 8% Bi_5_O_7_I/g-C_3_N_4_, respectively. The optimal degradation rate constant achieved by 8% Bi_5_O_7_I/g-C_3_N_4_ was approximately 9.45 times higher than that of pure g-C_3_N_4_, 4.26 times of pure BiOI and 2.40 times of pure Bi_5_O_7_I, respectively.

The photostability of 8% Bi_5_O_7_I/g-C_3_N_4_ was characterized through a five-cycle photocatalytic experiment, as shown in Figure 5C. No apparent deactivation was detected in five successive degradation reactions under visible light irradiation, suggesting the possibility for practical application in treatment of water pollution.

To investigate active species involved in the photocatalytic reaction, BQ, IPA and EDTA were used as scavengers to trap O_2_^−^, ·OH and h^+^, respectively. The effects of different scavengers on the removal efficiency of RhB were shown in Figure 5D. The results indicated that the removal percentage of RhB was significantly decreased to approximate 57, 38, 31% after addition of EDTA, BQ and IPA, respectively. These results revealed that all the reactive species of h^+^, ·OH, and O_2_^−^ participated in the photocatalytic degradation processes and all of them played important roles in the photocatalytic degradation reaction of RhB.

**FIGURE 6:**
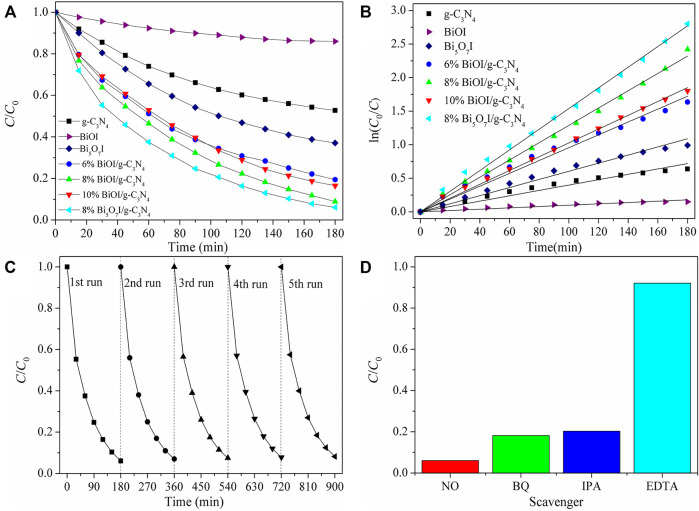
**(A)** Photocatalytic degradation of TCHbyas-prepared samples under visiblelight irradiation, **(B)** corresponding ln (*C*0*/C*) versus time of the degradation reaction, **(C)** cycling runs in the photocatalytic degradation of TCHwith 8% Bi5O7I/g-C3N4, **(D)** influence of different scavengers on degradation of TCH with 8% Bi5O7I/g-C3N4under visible light illumination after 180 min.

Figure 6 shows the photocatalytic activity of as-prepared samples in degradation of TCH under visible light illumination (≥420 nm). Similarly, 8% Bi_5_O_7_I/g-C_3_N_4_ achieved the superior photocatalytic activity. Photocatalytic activity descended in the queue as 8% Bi_5_O_7_I/g-C_3_N_4_> 8% BiOI/g-C_3_N_4_ > 10% BiOI/g-C_3_N_4_ > 6% BiOI/g-C_3_N_4_ > Bi_5_O_7_I > g-C_3_N_4_ > BiOI. Stability experiments indicated that sample 8% Bi_5_O_7_I/g-C_3_N_4_ showed a stable efficient photocatalytic performance in photodegradation of TCH. Scavengers experiments illustrated that h^+^ played an important role in the photocatalytic degradation reaction of TCH.

To further confirm complete photodegradation of RhB and TCH, COD of the solutions were measured before and after degradation tests. The COD values of the solutions before degradation of RhB and TCH were evaluated to be 9.5 mg/L and 32.0 mg/L respectively. Those after degradation of RhB and TCH were 1.2 mg/L and 2.8 mg/L, respectively, both of which were significantly lower than the WHO guideline value of 10 mg/L. It suggested very rare organic species in the solutions.

**FIGURE 7:**
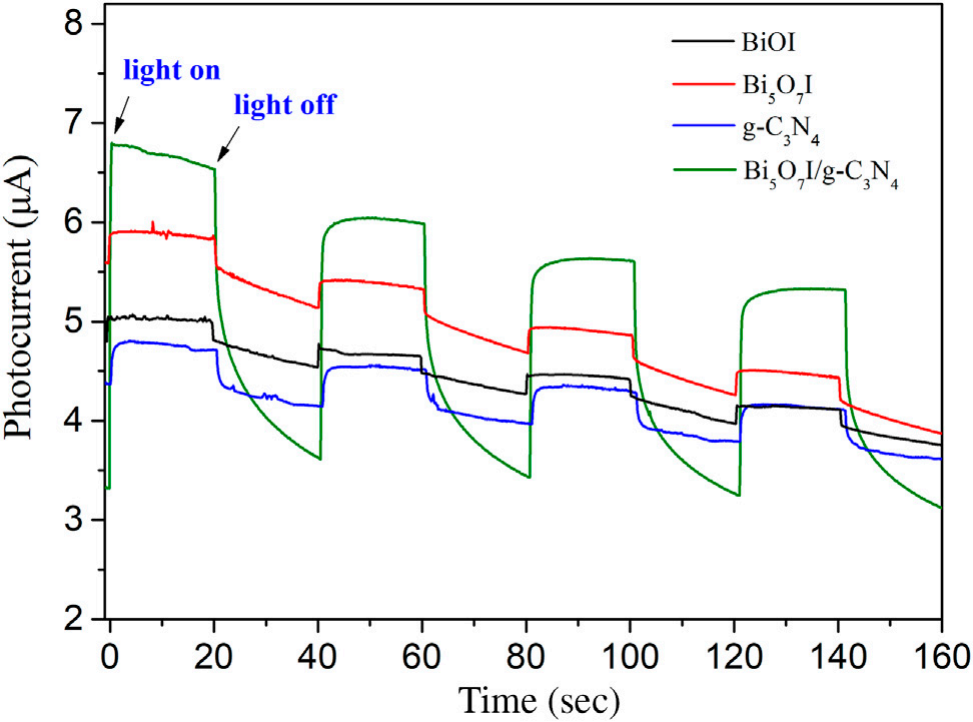
Comparison of transient photocurrent responses of pure BiOI, Bi5O7I, g-C3N4and g-C3N4/Bi5O7I composites with light on/off cycles under visible light irradiation.

Testing on transient photocurrent response were performed to study the transfer and separation efficiency of photogenerated charge carriers under visible light irradiation. Stronger photocurrent intensity usually indicates higher separation efficiency of holes and electrons. Figure 7 illustrates the transient photocurrent response curves of pure BiOI, Bi_5_O_7_I, g-C_3_N_4_ and Bi_5_O_7_I/g-C_3_N_4_ composite. Once the light source was turned on and off, the sample electrode exhibited a rapid photocurrent change. Furthermore, the Bi_5_O_7_I/g-C_3_N_4_ composite showed a strongest photocurrent response. It suggests that more efficient separation efficiency of electrons and holes occurred in Bi_5_O_7_I/g-C_3_N_4_ heterojunction, thus an enhanced photocatalytic activity was achieved by Bi_5_O_7_I/g-C_3_N_4_ composite photocatalyst.

### Proposed Mechanism for Enhanced Photocatalytic Activity With Bi_5_O_7_I/g-C_3_N_4_ Composites

**FIGURE 8:**
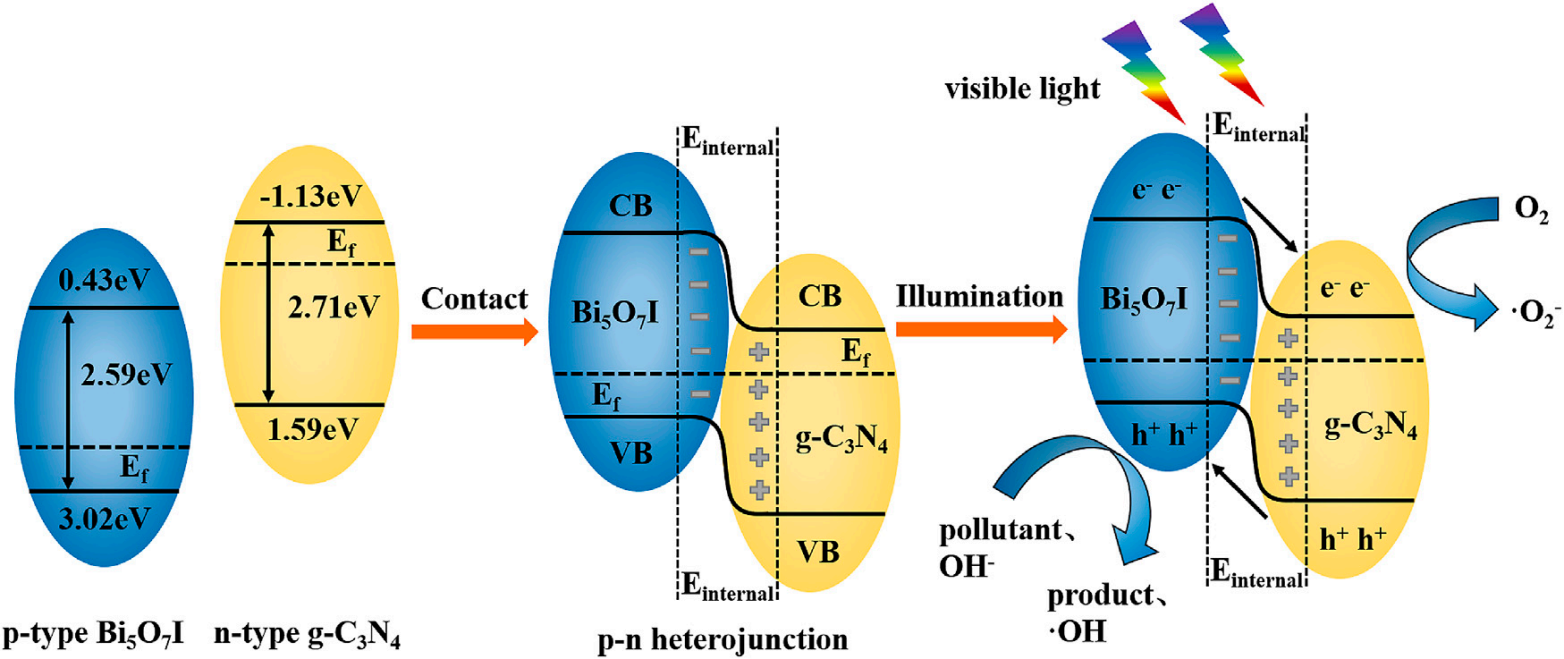
Proposed photocatalytic mechanism of Bi5O7I/g-C3N4.

Figure 8 shows the proposed photocatalytic mechanism of Bi_5_O_7_I/g-C_3_N_4_. The valence band edge potential and the conduction band edge potential of Bi_5_O_7_I and g-C_3_N_4_ were calculated using electronegativity with the following empirical equations (Li et al., 2015b)

$${\text{E}}_{\text{VB}}\;=\;\text{X}\;+\;\text{0}{\text{.}5\text{E}}_{\text{g}}\;-\;{\text{E}}_{\text{e}}$$ (2)

$${\text{E}}_{\text{CB}}\;=\;{\text{E}}_{\text{VB}}\;-\;{\text{E}}_{\text{g}}$$ (3)

$$\text{X}\;=\;{\left[x{\left(\text{A}\right)}^{\text{a}}x{\left(\text{B}\right)}^{\text{b}}x{\left(\text{C}\right)}^{\text{c}}\right]}^{1/\left(\text{a}+\text{b}+\text{c}\right)}$$ (4)

where *E*_VB_ is the valence band edge potential, *E*_CB_ is the conduction band edge potential, *x* is the electronegativity of chemical elements (a, b, and c are the atomic number of compounds), *E*_e_ is the energy of free electrons on the hydrogen scale (about 4.5 eV), *E*_g_ is the band-gap energy of semiconductor and *X* is the electronegativity of the semiconductor. The *X* value for Bi_5_O_7_I is about 6.22 eV. For g-C_3_N_4_, the *X* value is 4.73 eV (An et al., 2018). Therefore, the valence of *E*_VB_ and *E*_CB_ of Bi_5_O_7_I and g-C_3_N_4_ were calculated to be 3.02, 0.43 eV and 1.59, −1.13 eV, respectively. The Fermi energy (*E*_f_) level of Bi_5_O_7_I as a p-type semiconductor is closed to the valance band, while that of g-C_3_N_4_ as a n-type semiconductor is closed to the valence band. After contact was made between Bi_5_O_7_I and g-C_3_N_4_, electrons and holes migrated, i.e., electrons in g-C_3_N_4_ diffused to Bi_5_O_7_I and holes in Bi_5_O_7_I diffused to g-C_3_N_4_, which led to the formation of an electric field (Wang et al., 2014). The diffusion process continued until the Fermi levels of Bi_5_O_7_I and g-C_3_N_4_ reached an equilibrium state. Simultaneously, the valence and conduction bands of heterojunctioned Bi_5_O_7_I and g-C_3_N_4_ were bent to render a balanced state.

After visible light illumination, the induced holes from the valance band of g-C_3_N_4_ transferred to the valance band of Bi_5_O_7_I and the photoexcited electrons transferred from the conduction band of Bi_5_O_7_I to the conduction band of g-C_3_N_4_ driven by built-in electric field. It allows more effective separation and longer lifetime of photoinduced electron-hole pairs. Electrons gathered on the surface of g-C_3_N_4_ reacted with O_2_ in the water to form •O_2_^−^ and holes gathered on the surface of Bi_5_O_7_I reacted with OH^−^ in the water to form •OH (Lin et al., 2016), leading to the degradation of RhB. Simultaneously, holes on the valance band of Bi_5_O_7_I possesses a higher oxidation capability on RhB and TCH. The formation of Bi_5_O_7_I/g-C_3_N_4_ heterojunction not only expands the photoresponse region, but also suppresses the recombination of photogenerated electron-hole pairs and improves the photocatalytic activity.

## Conclusion

In summary, visible-light-driven heterostructured Bi_5_O_7_I/g-C_3_N_4_ photocatalysts were designed and synthesized by alcohol-heating and calcination processes. Enhanced photodegradation performance of RhB and TCH under visible light (λ> 420 nm) was achieved by the 8% Bi_5_O_7_I/g-C_3_N_4_ composite compared with pure BiOI, g-C_3_N_4_ and Bi_5_O_7_I. An effective built-in electric field was formed by the interface between p-type Bi_5_O_7_I and n-type g-C_3_N_4_, which promoted the efficient separation of photogenerated electron-hole pairs. Furthermore, 8% Bi_5_O_7_I/g-C_3_N_4_ composite showed excellent photostability in a five-cycle photocatalytic experiment. Experiments on scavenging active intermediates revealed that h^+^, ·OH, and O_2_^−^ were all active species in photodegradation of RhB and h^+^ was dominant in photodegradation of TCH.

## Data Availability

The original contributions presented in the study are included in the article/Supplementary Material, further inquiries can be directed to the corresponding authors.
