# Supplementary figures and images for: Bi5O7I/g-C3N4 Heterostructures With Enhanced Visible-Light Photocatalytic Performance for Degradation of Tetracycline Hydrochloride
Source: Front Chem. 2021 Dec 14;9:781991. doi: 10.3389/fchem.2021.781991 (PMC8712322; doi:10.3389/fchem.2021.781991)

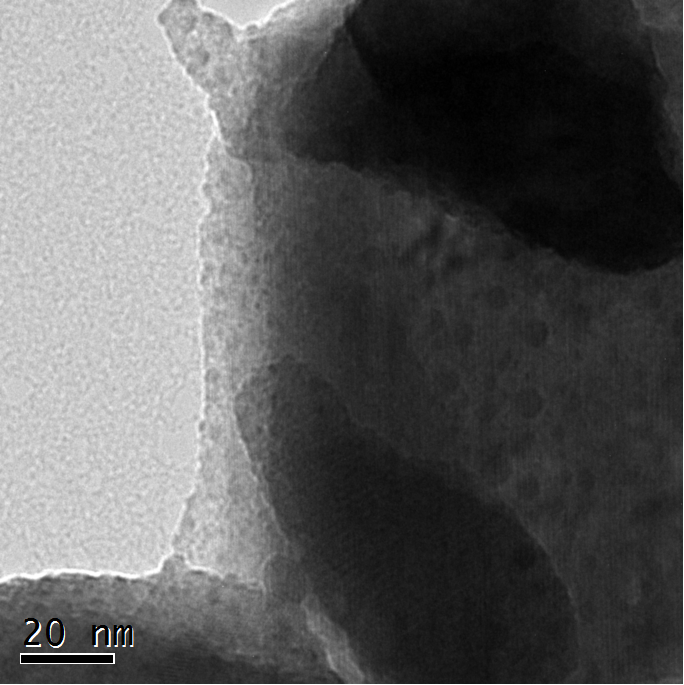

Supplement: Supplementary file 3 [file Image6.TIF]

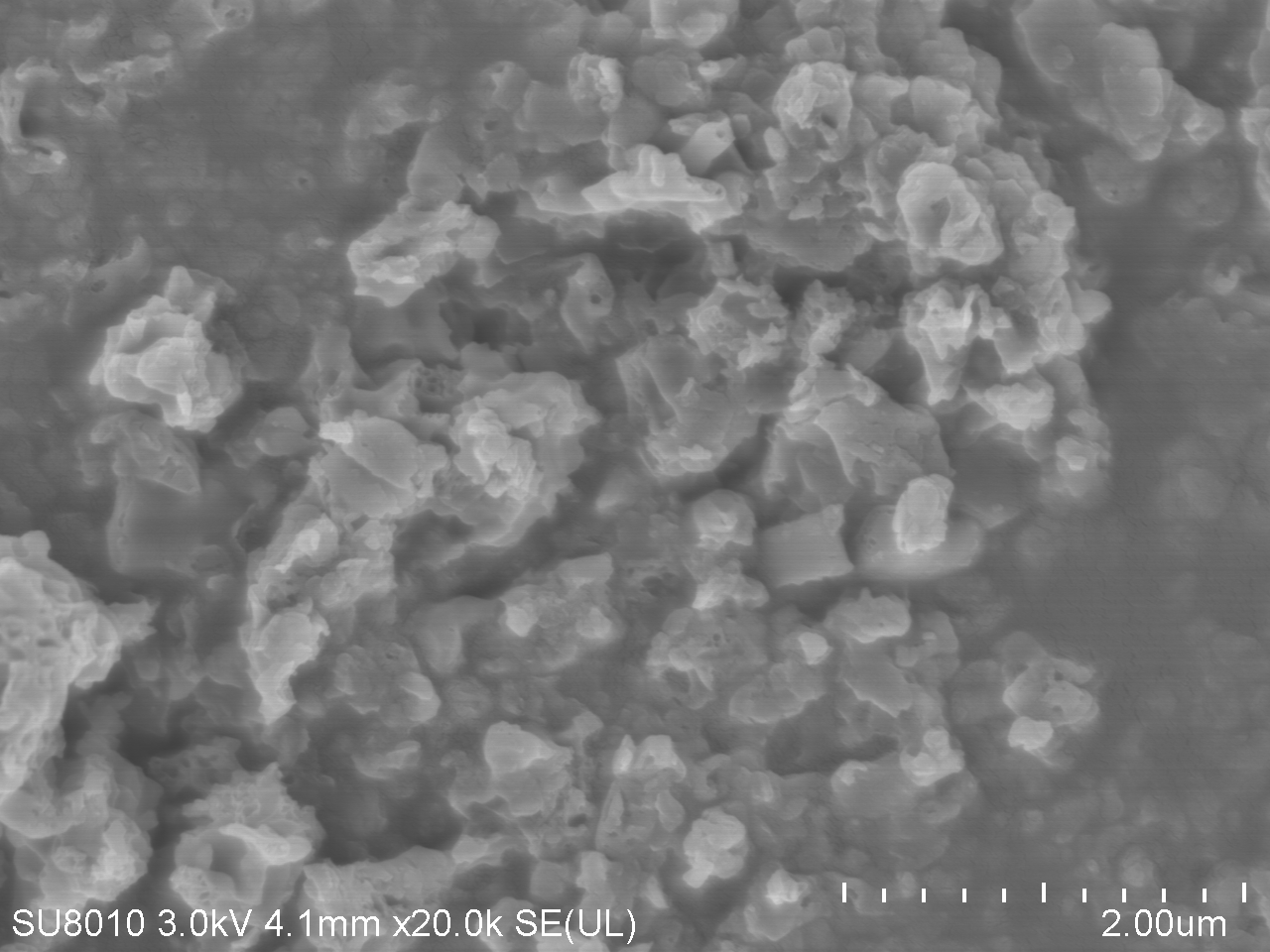

Supplement: Supplementary file 4 [file Image3.TIF]

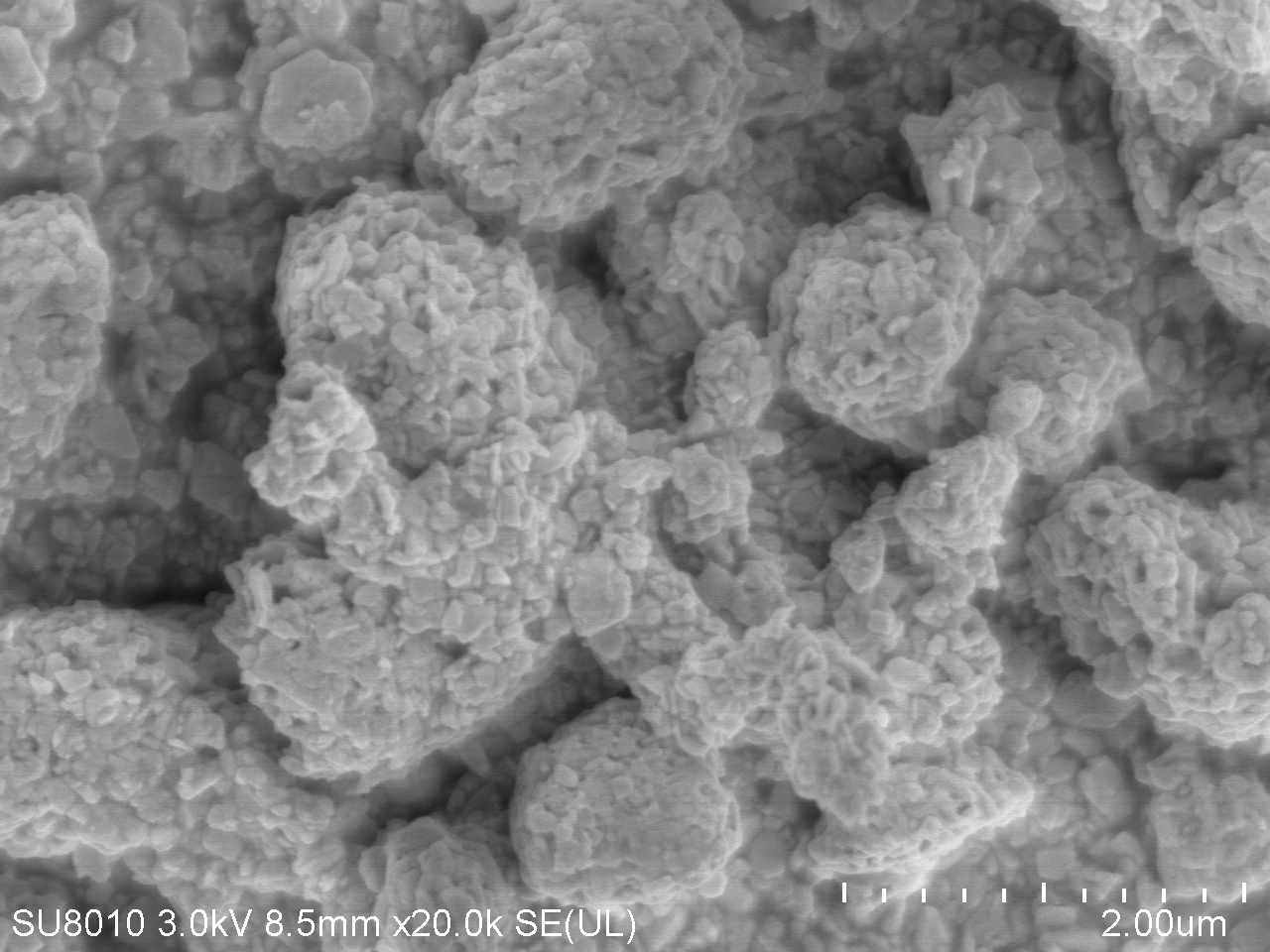

Supplement: Supplementary file 5 [file Image4.TIF]

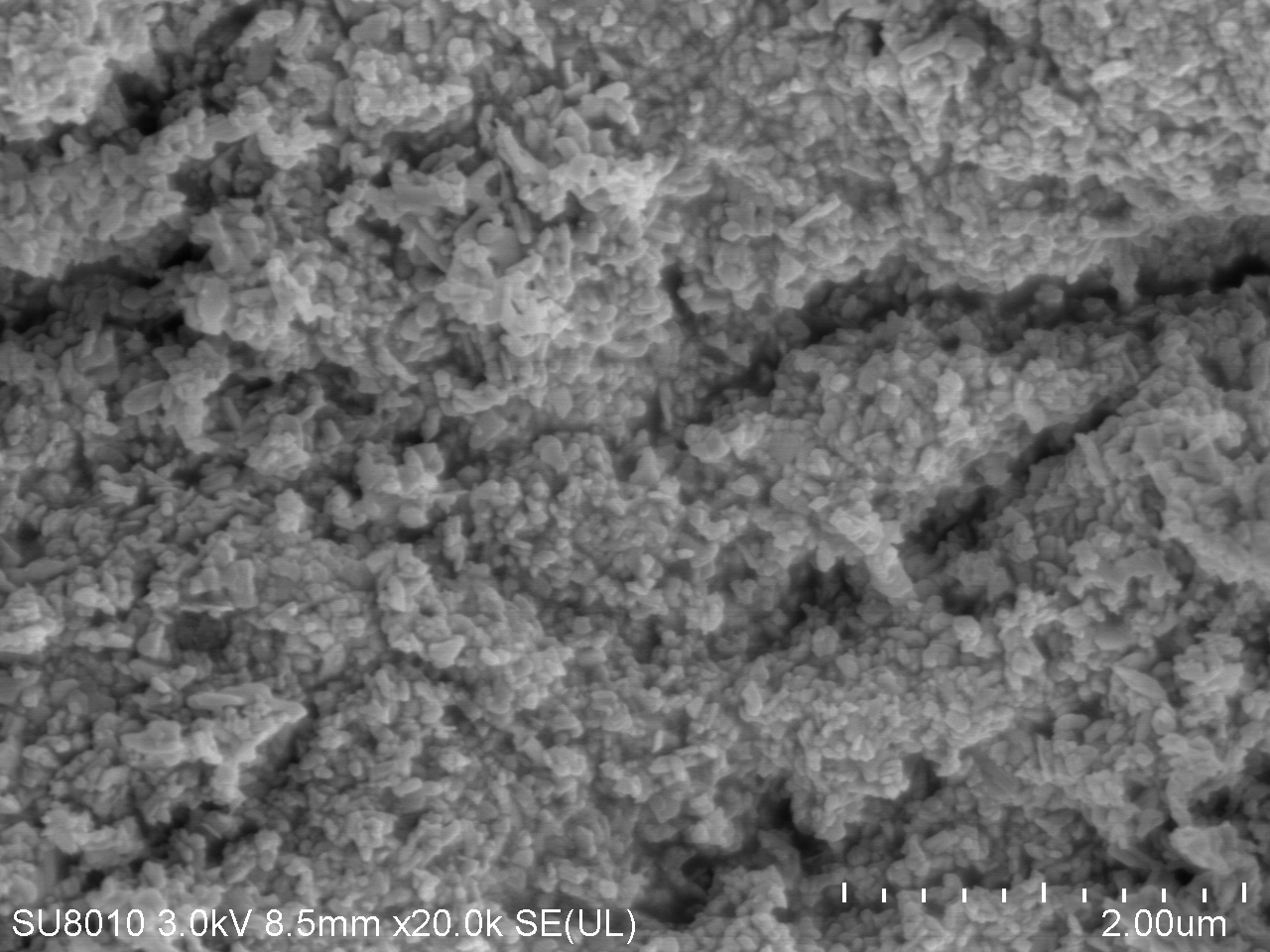

Supplement: Supplementary file 6 [file Image2.TIF]

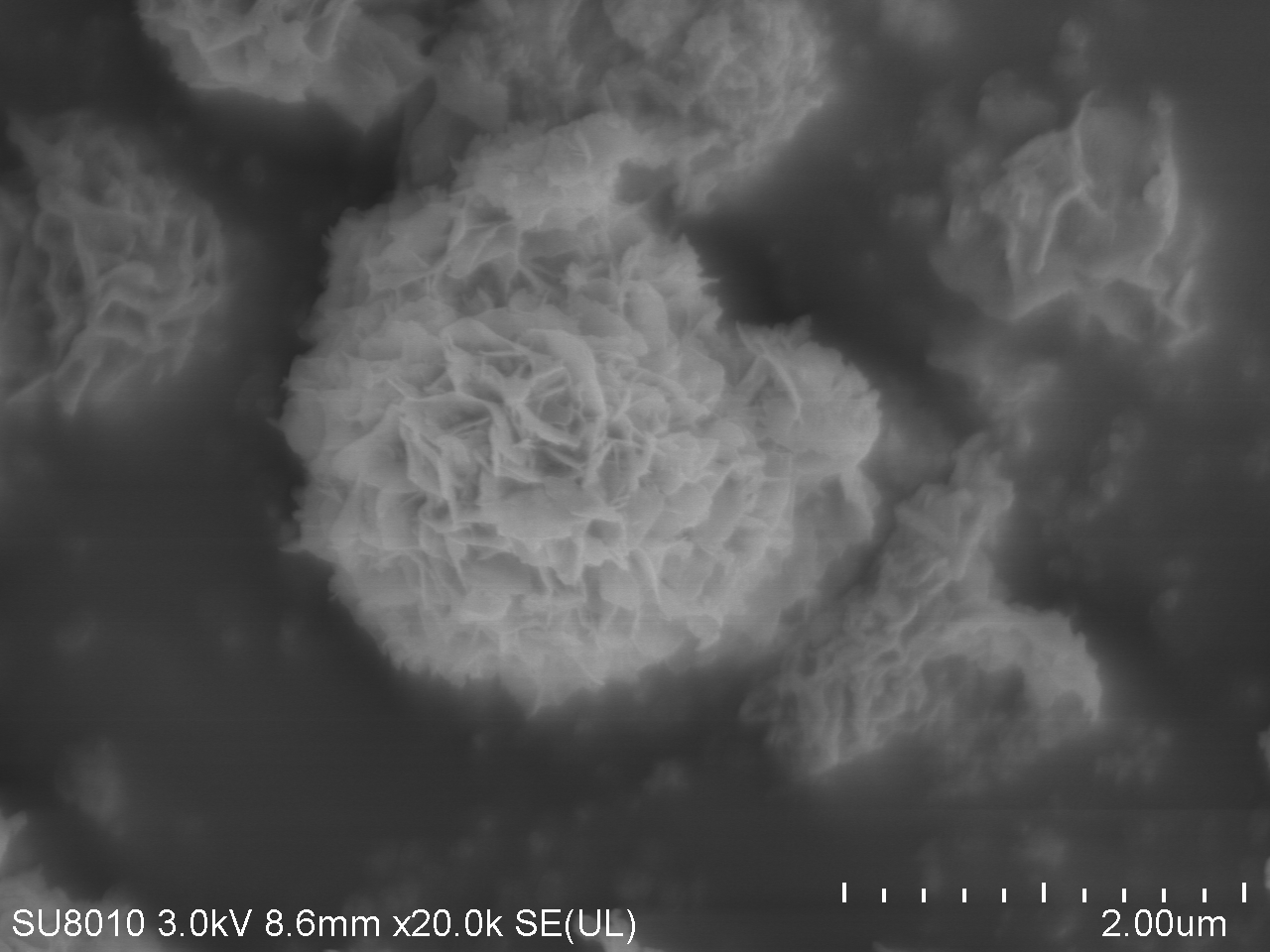

Supplement: Supplementary file 7 [file Image1.TIF]

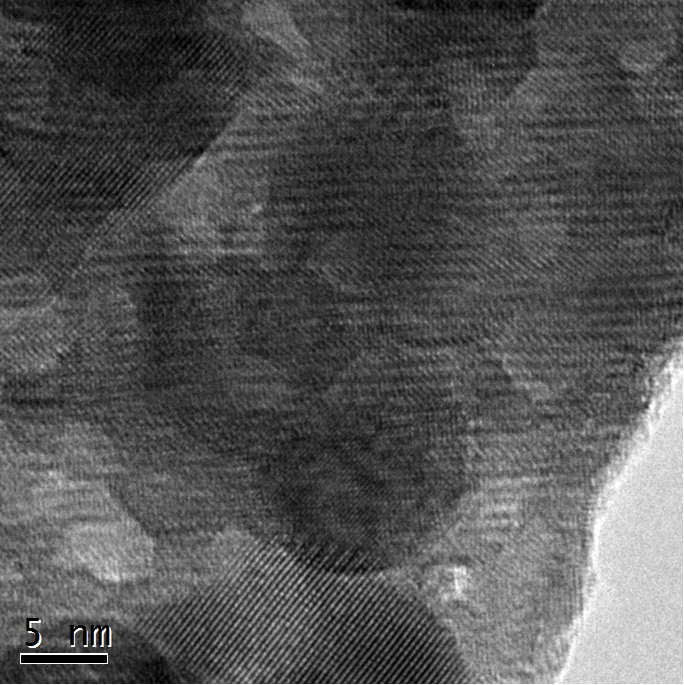

Supplement: Supplementary file 8 [file Image7.TIF]

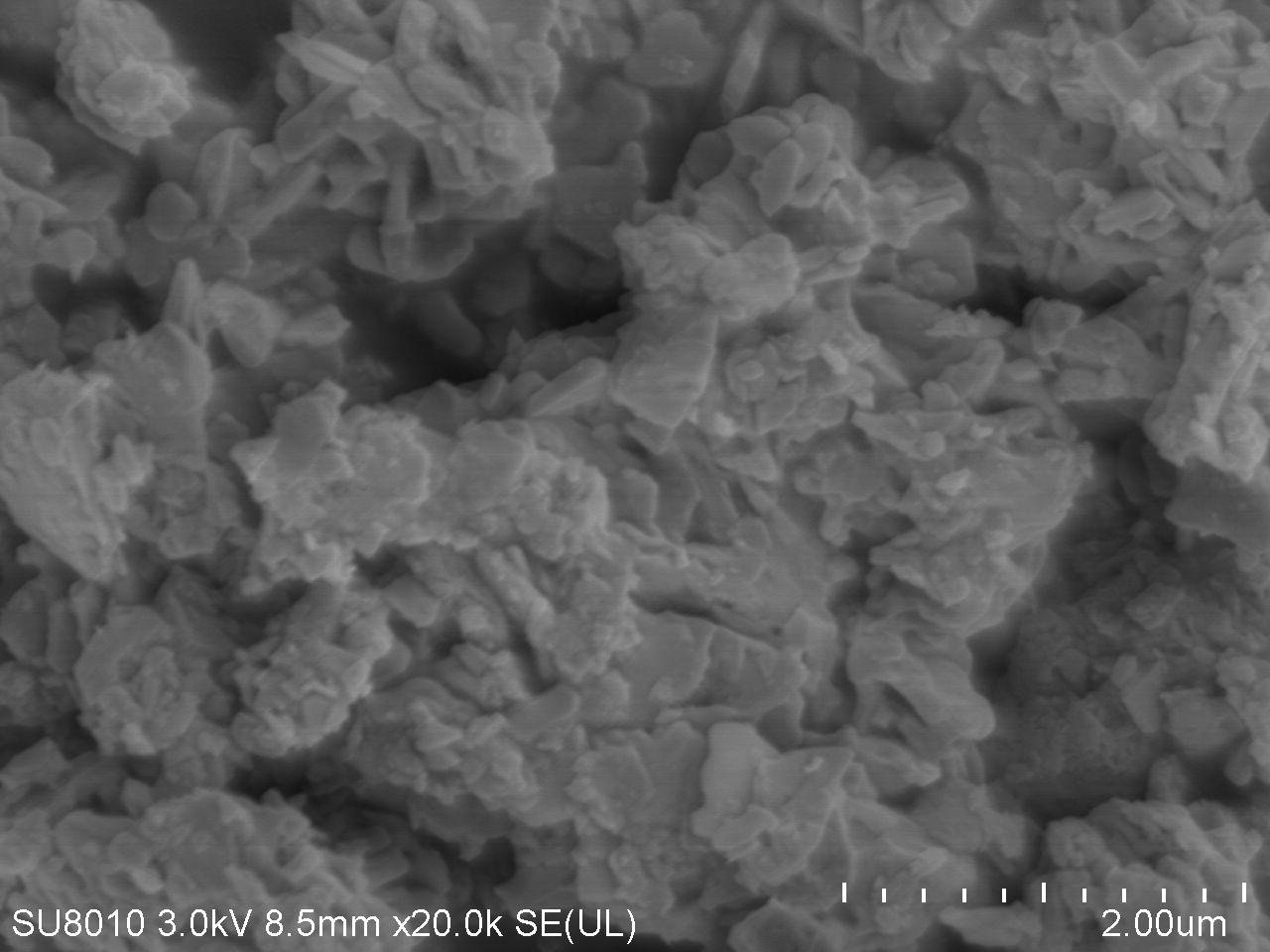

Supplement: Supplementary file 11 [file Image5.TIF]
